# Article visibility: journal impact factor and availability of full text in PubMed Central and open access

**Published:** 2013-10

**Authors:** Paul A Brink

**Affiliations:** Department of Internal Medicine, Faculty of Health Sciences, University of Stellenbosch and Tygerberg Hospital, Tygerberg

Both the impact factor of the journal and immediate full-text availability in Pubmed Central (PMC) have featured in editorials before.^1^-^3^ In 2004, the editor of the *Cardiovascular Journal of Africa* (CVJA) lamented, like so many others, the injustice of not having an impact factor, its validity as a tool for measuring science output, and the negative effect of a low perceived impact in drawing attention from publications from developing countries.^1^,^4^

Since then, after a selection process, we have been indexed by the Web of Science® (WoS) and Thomson Reuters (Philadelphia, PA, USA), and have seen a growing impact factor. In the case of PMC, our acceptance to this database was announced in 2012,^2^ and now we are proud that it is active and full-text articles are available dating back to 2009. The journal opted for immediate full open access (OA), which means that full-text articles are available on publication date for anybody with access to the internet.

## The journal impact factor (JIF)

The impact factor is one measurement of visibility of articles in specific journals and is more appropriately called the journal impact factor (JIF). It was originally developed by Eugene Garfield as a help to librarians in selecting journals to which to subscribe.^5^,^6^ However, it acquired iconic status as a single measure of the quality of science published in a journal and by extension, the scientific standing of authors, affecting, among others, grant allocation and career advancement.

The classic JIF is defined as the ratio of the number of cites in a given year as a ratio of the number of ‘citable’ articles published in the previous two years. Information comes from the approximately 11 000 journals indexed by the WoS, which is published in the Journal Citation Report® (JCR).^5^,^6^ Cites, counted in the numerator, can be from any type of article from journals within the database, whereas only articles designated as research or review count in the denominator.

Factors that can be correlated with the JIF have been much researched and debated. To name but a few, the country of the journal and its socio-economic status,^7^ the field of publishing, number or reviews versus original research articles, and the effect of a blockbuster article.^5^,^6^

The *New England Journal of Medicine* (NEJM) with a JIF of 52.658 (JCR 2012) and then Lancet with a JIF of 39.060 (2012) are probably the world’s most read medical journals. South Africa’s general medical journal, the *South Africa Medical Journal*, comes in at a JIF of 1.702.

Surprisingly, the highest JIF belongs to a journal that most readers of CVJA may not even have heard off, a clinical oncology journal, *Cancer Journal for Clinicians*, with a JIF of 153.459 (2012). It publishes few articles, 37 in 2010 and 11, but with 5 678 cites in 2012. It went from a JIF of 101.78 in 2011 to 153.459 in 2012, the highest jump ever recorded. This can mostly be attributed to an extraordinary number of cites, 1 787 to one article (26% of cites).^8^ From this, one can calculate a contribution to the JIF of 39.945, which is more than the JIF of the *Lancet*, which had 21 366 cites for 547 articles, translating into its JIF of 39.060.

Of course, when working in the cardiovascular field, one will not publish in a cancer journal. In the JCR field of cardiac and cardiovascular systems, where the CVJA resides, *Circulation* is top, with a JIF of 15.202. However, the CVJA is from Africa. Of the 46 African countries in the JCR, a non-medical journal the *International Journal of Photoenergy* from Egypt but domiciled in New York, has the highest JIF of 2.663. Second is the *South African Medical Journal*, which forms part of the medical group of 14 African journals within the JCR, with a JIF of 1.702. Of these, the CVJA with a JIF of 0.848 ranks a close third after the *African Journal of Psychiatry*, with a JIF of 0.871. Within the 14 titles, CVJA is the only journal from Africa in the field of cardiac and cardiovascular systems.

Having publications accepted in journals with a high JIF does not necessarily reflect an interest in local problems, but often current interest in developed economies. For example, in the NEJM over the eight-year period from 1997 to 2004, less than 3% of published articles addressed health issues of developing countries. Furthermore, there was a bias towards HIV/AIDS and infective disease. Of 202 articles addressing issues of developing countries, 135 (almost 50% HIV/AIDS related) were about infectious disease. Only 23 were about non-communicable disease, of which one, a book review, discussed heart disease.^9^ So, in principle, it is possible that exhortation to publish in highimpact journals, as is often the practice, may skew research to improve visibility in the developed world.

Then there is also the excessive emphasis on a single metric, the ‘number that’s devoured science’, the JIF.^10^ Who in clinical medicine relies on a single sign or a single test to make a diagnosis? The astute physician usually considers a constellation of findings and tests in coming to a diagnostic conclusion. This should be the same in the bibliometric evaluation of science, and in the San Francisco Declaration on Research Assessment, a group of editors and publishers made a plea for a more broadbased approach.^11^

The group suggests a reduction in emphasis on the journal impact factor and to create context by using a variety of journal-based metrics, e.g. five-year impact factor, EigenFactor, SCImago, h-index, editorial and publication times, all of which are available for the CVJA. A description of these metrics, their use and citation databases other than WoS, such as Scopus and Google Scholar can be read in Bornman, *et al.* and Pendlebury.^5^,^6^ The group also recommends consideration of non-journal factors, such as effect on health policy and to develop metrics for measuring scientific content rather than only publication metrics.

## The journal and Pubmed Central

With CVJA, the other important development has been listing in PMC. PMC is a free full-text archive of biomedical and life sciences journal literature at the US National Institutes of Health’s National Library of Medicine (NIH/NLM). Advantages and disadvantages have been discussed.^2^,^12^ One can access PMC-listed CVJA articles directly (http://www.ncbi.nlm.nih.gov/pmc/journals/1961/) where all the issues from 2009 to current are available in three formats: HTML, PDF and tablet friendly, the latter very user friendly. Note that only primary research and review articles are listed on PMC.

In a sense, one is using more than a belt and braces in using multiple repositories. This ensures that articles published in the journal will survive for a long time in cyberspace. The same articles, in addition to editorial and other content in the journal, can also be accessed through Pubmed (http://www.ncbi.nlm.nih.gov/pubmed) by an article search. Here one can access full text in addition to case studies published online through SAePub (Sabinet).

Then there is the website of CVJA (http://www.cvja.co.za/) where one can read articles in a number of formats, including an e-reader format or, even better, browse through a facsimile of the journal as it appears in print, with everything, adverts included. Advertising income plays an important role in sustainability of the CVJA and it is therefore important to maximise traffic through the website of the CVJA.

In this regard, PMC has been shown to divert traffic from PMC-listed journals’ websites or other repositories.^12^ The ability to cross-reference data from diverse sources, clinical, genetic, DNA sequence, and protein is potentially very useful. For example, accessing a recent article on long QT syndrome brings up references to DNA sequences coding for cardiac ion channels (a cause of LQTS) and also information on the channel proteins contained within the NIH/NLM databases.^13^ However, this easy networking within PMC may create less of an impetus to use the website of the CVJA.

In accessing OA articles, albeit through the journals’ websites, Pubmed, PMC or Sabinet, one needs only access to the internet. This is good news for readers from low-resource settings. No personal, departmental and library subscription is necessary. There is however no such thing as a free lunch. The publication bill needs to be settled.

With OA, distribution costs can be very low if a journal chooses to publish only online, but there are still high costs involved for proper peer review and editorial quality control. Production costs are not necessarily cheaper and cost falls to the author or institution.^12^,^14^ For example, publishing in *PLOS Medicine*, an OA journal with high impact, it will cost the author(s) $1 900 (graded according to the economic status of a country). In the case of CVJA, the up-front cost is $50 to have a submission reviewed and the rest of the costs are covered by advertisements, special projects and subscriptions. As indicated, however, the last modality may be under threat. For the other sources of income, using the CVJA website as a portal of entry is very important.

It may well happen that institutions limit subscriptions to journals not OA but deemed very important, such as *Circulation* and NEJM. It is interesting that a number of high-impact journals with content perceived to be commercially of value have not embraced PMC or OA, such as the NEJM and *Circulation*. Other funds may be needed to carry publication costs on the authorpay principle. All research is not backed by strong institutional funding, especially not in Africa. This does provide challenges!

## Conclusion

The CVJA is from Africa, but also part of the global environment. Within Africa, the journal is well placed and a portal to the world. Articles within the journal are visible, as the journal is indexed in the major databases. Content is freely available on date of publication, but the OA environment has created new challenges. A last thought: Is there not a place for an African journals database so that we can unlock synergies within Africa?

## References

[1] Brink AJ (2004). Impact factor: use and abuse.. Cardiovasc J S Afr.

[2] Brink AJ (2012). New impact factor and PubMed Central service from the Cardiovascular Journal of Africa.. Cardiovasc J Afr.

[3] Brink AJ (2012). Electronic innovation and readership.. Cardiovasc J Afr.

[4] Rossner M, Van EH, Hill E (2008). Show me the data.. Cardiovasc J Afr.

[5] Bornmann L, Marx W, Gasparyan AY, Kitas GD (2012). Diversity, value and limitations of the journal impact factor and alternative metrics.. Rheumatol Int.

[6] Pendlebury DA (2009). The use and misuse of journal metrics and other citation indicators.. Arch Immunol Ther Exp (Warsz ).

[7] Yousefi-Nooraie R, Shakiba B, Mortaz-Hejri S (2006). Country development and manuscript selection bias: a review of published studies.. BMC Med Res Methodol.

[8] Jemal A, Bray F, Center MM, Ferlay J, Ward E, Forman D (2011). Global cancer statistics.. CA Cancer J Clin.

[9] Lown B, Banerjee A (2006). The developing world in the New England Journal of Medicine.. Globalization Health.

[10] Monastersky R (2005). The number that’s devouring science (grading system for scholarly journals).. Chron Higher Ed.

[11] Way M, Ahmad SA (2013). The San Francisco Declaration on Research Assessment.. J Cell Sci.

[12] Frank M (2013). Open but not free – publishing in the 21st Century.. New Engl J Med.

[13] Hedley P, Durrheim G, Hendricks F (2013). Long QT syndrome in South Africa: the results of comprehensive genetic screening.. Cardiovasc J Afr.

[14] Haug C (2013). The Downside of Open-Access Publishing.. New Engl J Med.

